# Barriers and Facilitators of Taking a Lifestyle History and Referral to Lifestyle Interventions in Mental Health

**DOI:** 10.1177/15598276241261670

**Published:** 2024-06-13

**Authors:** L. E. M. Koomen, J. Deenik, W. Cahn

**Affiliations:** 1 8124 UMC Utrecht, Utrecht, The Netherlands (LEMK); 2 GGz Centraal, Maastricht University, Amersfoort, The Netherlands (JD); 3 Altrecht Mental Health Institute, 8124 UMC Utrecht, Utrecht, The Netherlands (WC)

**Keywords:** lifestyle psychiatry, implementation, physical health, barriers, facilitators, cross-sectional study

## Abstract

Background: Lifestyle interventions can improve mental and physical health in patients with mental illness, but implementing these in clinical practice seems difficult. Purpose: Investigate barriers and facilitators for mental health professionals (MHPs) in taking lifestyle histories and referring to lifestyle interventions. Methods: A cross-sectional national online survey among MHPs. All mental health care institutions, hospital psychiatry departments, associations for nurse specialists, and independent working psychiatrists’ organizations in the Netherlands were invited to participate. Ordinal regression analyses were performed to study factors associated with barriers. Results: 1524 MHPs participated. Barriers were time constraints (45.3%), lack of referral possibilities (33.2%), patient disinterest (25.4%) and lack of knowledge about: effect (25.5%), availability of interventions (57.5%), lifestyle (16.9%), and reimbursement (41.5%). Facilitators included more referral possibilities (44.9%), integration of lifestyle into clinical routine (48.3%), a dedicated tool (41.5%), organizational commitment (41.2%) and lifestyle as standard treatment component (40.3%), and more knowledge about: referral possibilities (51.4%), effect (38.1%), and reimbursement (48.1%). Older MHPs, those who consider their own lifestyle important, and those working in organizations where lifestyle interventions are available experienced fewer barriers. Conclusions: Organizations should prioritize lifestyle psychiatry by educating staff, integration into clinical routine, and increasing the availability and reimbursements of interventions.

“Identified barriers included time constraints, limited referral options, patient disinterest, and lack of knowledge about intervention effects, availability, lifestyle, and reimbursement ”

## Introduction

People with mental illness face severe health challenges. They often experience long-lasting psychiatric symptoms, resulting in lower quality of life. They are also at increased risk for cardiometabolic diseases and premature mortality.^7,10,11^ Lifestyle interventions, such as increasing physical activity (e.g., running therapy), improving dietary habits, insomnia cognitive behavioral to improve sleep, and promoting smoking cessation, can improve mental and physical health across a range of diagnoses.^5,6^ Therefore, these interventions are recommended by international guidelines.^12,13,19^

However, implementing lifestyle interventions in clinical practice is challenging,^3,5^ and it is still not common practice to take lifestyle histories and refer patients to lifestyle interventions. In the first evaluation of this study on 1607 Dutch mental health professionals (MHPs), we found that depending on the lifestyle factor (physical activity, dietary habits, sleep, smoking, and drinking habits), 55.1%-84.0% of MHPs report taking a lifestyle history. Moreover, 41.1% of the patients are referred to lifestyle interventions to improve mental health, and 37.9% to improve physical health.^9^ In Australia, exercise was prescribed daily or weekly for depression, anxiety, or stress by 70% of the MHPs, but only by 11% of the MHPs for schizophrenia or bipolar disorder.^15^

MHPs face barriers that hinder them from taking lifestyle histories and referring patients to lifestyle interventions. For exercise prescription, identified barriers include patient disinclination, patient mental health problems, lack of knowledge, concern about patient injuries, and the belief that it is not the MHP’s responsibility.^8,15^ A small study in Uganda found that nurses’ high workload is also a barrier.^14^ In general health care, HCPs found referral to lifestyle interventions to be a non-essential part of their work. Barriers include poor communication with programs, lack of training, limited reimbursement, skepticism about the effect, patient disinterest, and unclear lines about responsibility for patient care once they were referred.^18^ Facilitators were access to education, awareness of interventions, interdisciplinary collaborations, a broad range of accessible interventions, and easy referral.^18^

While barriers and facilitators were often investigated in general health care,^18^ only 3 studies^8,14,15^ focused on mental health care, primarily on exercise prescription. Studies highlight the need for more knowledge on barriers and facilitators faced by MHPs regarding clinical lifestyle practices to improve the implementation of lifestyle interventions.^3,5,13^ Therefore, this cross-sectional survey aimed to examine the barriers and facilitators perceived by MHPs regarding taking lifestyle histories and referring patients to lifestyle interventions, and to assess whether these barriers are associated with MHPs’ lifestyle habits, profession, and the availability of interventions. This study is an extension of a previously published study on Dutch MHP’s clinical lifestyle practices, and there is no overlap in the reported data.^9^

## Methods

This cross-sectional study was performed from May 2022 until January 2023. According to Dutch law, medical ethical approval is not required for non-medical research involving anonymous data. The rules of conduct as outlined in the Declaration of Helsinki were followed, and participants were asked for consent. This study was reported according to the STROBE guideline.^4^

### Sample and Recruitment

All mental health care institutions, (academic) hospital psychiatry departments, associations for nurse specialists, organizations for GP-based nurse specialists, and independent working psychiatrists’ organizations in the Netherlands were invited to share the online questionnaire link with referring MHPs. Furthermore, invitations to participate in the online survey were disseminated during the Dutch Association for Psychiatry (NVvP) 2022 congress, the primary psychiatry conference in the country.

Referring MHPs were recruited for participation, including residents in psychiatry, general-practice (GP) based nurse specialists, MHPs with a nursing background (social psychiatric nurses, nurse specialists, physician assistants), MHPs with a scientific background (health care psychologists, remedial educationalists, psychotherapists), clinical psychologists, and psychiatrists. Non-referring MHPs (e.g., psychologists, nurses, psychomotor therapists) who filled in the survey were included in the study but excluded from the analysis on referring practices.

All participants who filled in the entire questionnaire were included in this study. Therefore, the sample size of this study differs from the first evaluation of the study (1524 vs 1607), in which participants were included if they completed the relevant questions for that evaluation.^9^

### Measurements

The study used a self-report online survey consisting of open and closed-ended questions on demographics, work setting, lifestyle habits, and questions regarding barriers and facilitators of taking a lifestyle history on alcohol and tobacco use, physical activity, dietary habits, and sleep, as well as referral to lifestyle interventions (Supplementary Material and previous study^9^). Questions regarding physical activity were based on the international physical activity guidelines: ≥2x bone and muscle strengthening exercises per week, and ≥150 min moderate to vigorous physical activity (MVPA) per week.^1^ Questions about tobacco use and drinking habits were based on the FANTASTIC instrument.^17^

### Data Analysis

For all answers numbers, percentages, means and standard deviations were calculated. Responses to open-ended questions were manually assessed and listed in Table S3. Barriers were categorized, and an ordinal regression was performed including the following variables: availability of lifestyle interventions at the organization, age (per 10 years), gender, body mass index (BMI) in kg/m^2^ (per 5 units), importance of own lifestyle (0-10), smoking status, alcohol use, adherence to Dutch physical activity guidelines (=≥2x bone and muscle strengthening exercises per week, and ≥150 min moderate to vigorous physical activity per week),^2^ balanced eating pattern, sleep satisfaction, and profession. SPSS version 27.0 was used. A Bonferroni correction was done to account for multiple testing as seven ordinal regression analyses were conducted. A *P*-value of .007 was considered statistically significant.

## Results

A total of 1524 MHPs participated (Table S2). Participants had an average age of 42.2 years (SD = 11.3), with 78.6% (N = 1198) being female, which is a typical representation of Dutch mental health care workers.^16^ The sample included 329 (21.6%) referring MHPs with a scientific background, 285 (18.7%) psychiatrists, 225 (14.8%) referring MHPs with a nursing background, 191 (12.5%) GP-based nurse specialists, 168 (11.0%) psychologists, 156 (10.2%) residents in psychiatry, 81 (5.3%) clinical psychologists. Most participants worked in a mental health organization (72.9%, N = 1111), followed by GP practices (12.0%, N = 183), and worked with adult patients (65.9%, N = 1004). Lifestyle interventions were available at 63.3% (N = 965) of the organizations, and 87.8% (N = 1338) agreed that lifestyle should be part of every psychiatric treatment.

### Barriers and Facilitators for Taking a Lifestyle History

Table 1 shows an overview of the top five barriers and facilitators of taking a lifestyle history and referral to lifestyle interventions. A minority of participants (17.7%, n = 270) did not experience barriers to taking a lifestyle history. Overshadowing of patient’s mental problems (n = 52, 25.1% of the open-ended question) and forgetting to take a lifestyle history (n = 26, 12.6%) were identified as barriers in the open-ended question (Table S3). “Overshadowing of mental problems” refers to situations where psychiatric symptoms are so prominent that other issues receive less or no attention. Facilitators included having more interventions available in their organization (n = 45, 17.9%), and using a structured method for taking a lifestyle-history, diagnostics, treatment, and embedding in meetings (n = 45, 17.9%).

**Table 1.** Barriers and Facilitators of Taking a Lifestyle History and Referral to Lifestyle Interventions. N = number.

Barriers	N (%)	Facilitators	N (%)
Taking a Lifestyle History			
Lack of time	690 (45.3)	More knowledge about referral possibilities	783 (51.4)
Lack of knowledge about referral possibilities	616 (40.4)	Integrating lifestyle in daily working routines	736 (48.3)
Lack of knowledge about the effect of lifestyle on mental health	389 (25.5)	A dedicated tool (e.g., questionnaire, app)	632 (41.5)
Patients do not find it important	387 (25.4)	It would help if it is an organization’s goal	628 (41.2)
Lack of knowledge about lifestyle	258 (16.9)	More knowledge about the effect of lifestyle on mental health	581 (38.1)
Referral to lifestyle interventions			
Lack of knowledge about referral possibilities	724 (57.5)	More knowledge about referral possibilities	778 (61.8)
Lack of knowledge about reimbursement of lifestyle interventions	522 (41.5)	More knowledge about reimbursement of lifestyle interventions	606 (48.1)
Lack of referral possibilities within their organization	418 (33.2)	More referral possibilities within their organization	565 (44.9)
Lack of time	368 (29.2)	More referral possibilities within the region	528 (41.9)
Lack of referral possibilities within the region	332 (26.4)	It would help if lifestyle interventions are standard in the treatment within their organization	507 (40.3)

Ordinal regression analysis (Table S4) showed that older MHPs experienced fewer organizational barriers (OR = .75|CI = .61-.93), as did those with higher sleep satisfaction (OR = .92|CI = .86-.97), and those working in organizations where interventions were available (OR = .74|CI = .61-.93). Conversely, more organizational barriers were reported by MHPs who were more physically active (OR = 1.48|CI = 1.13-1.94). Older MHPs experienced fewer knowledge barriers (OR = .68|CI = .61-.76), as did those who find their lifestyle important (OR = .83|CI = .76-.92), and those working in organizations with available interventions (OR = .65|CI = .53-.79). The barrier patient disinterest was less experienced by older MHPs (OR = .82|CI = .72-.93). MHPs’ personal lifestyle was less of a barrier for older MHPs (OR = .59|CI = .40-.86), and those who find their lifestyle important (OR = .60|CI = .46-.80), but more of a barrier for MHPs with a higher BMI (OR = 2.99|CI = 2.10-4.27). Compared to psychiatrists, psychologists, GP-based nurse specialists, and MHPs with a scientific background experienced fewer organizational barriers (OR = .50|CI = .33-.76 | OR = .36|CI = .25 .53 | OR = .54|CI = .39-.75). MHPs with a nursing background experience fewer knowledge barriers (OR = .58|CI = .41-.82) than psychiatrists.

Compared to psychiatrists, psychologists, GP-based nurse specialists, and MHPs with a scientific background experienced fewer organizational barriers (OR = .50|CI = .33-.76 | OR = .36|CI = .25-.53 | OR = .54|CI = .39-.75). Additionally, MHPs with a nursing background experienced fewer knowledge barriers (OR = .58|CI = .41-.82) than psychiatrists.

### Barriers and Facilitators for Referral to Lifestyle Interventions

The majority of MHPs (87.0%, n = 1099) experienced barriers for referral to interventions. The main barrier was a lack of knowledge about referral possibilities, while the key facilitator was, unsurprisingly, having more knowledge about referral options. Lack of reimbursement (n = 13, 24.5%) and lack of patient motivation (n = 12, 22.6%) were identified as barriers in the open-ended question. Facilitators included the availability of more accessible interventions (n = 13, 22.0%) and societal prioritization of a healthy lifestyle (n = 7, 11.9%).

Ordinal regression (Table S5) showed that fewer organizational barriers were experienced by older MHPs (Or = .77|CI = .69-.87), as well as by GP-based nurse specialists (OR = .32|CI = .22-.45) and MHPs with a scientific background compared to psychiatrists (OR = .51|CI = .37-.70). Older MHPs (OR = .68|CI = .55-.85) and those working in organizations with interventions available (OR = .73|CI = .65-.81) experienced fewer knowledge barriers.

Personal lifestyle habits were less of a barrier for MHPs who considered their lifestyle important (OR = .48|CI = .31-.72) and those who never smoked (OR = .11|CI = .03-.43), but more for MHPs with a higher BMI (OR = 2.49|CI = 1.46-4.25).

## Discussion

This study aimed to examine barriers and facilitators of clinical lifestyle practices and assess if MHPs’ characteristics and the availability of lifestyle interventions were related to their perceived barriers. Identified barriers included time constraints, limited referral options, patient disinterest, and lack of knowledge about intervention effects, availability, lifestyle, and reimbursement. Facilitators included more referral options, integration of lifestyle into clinical routines, a dedicated tool, prioritizing lifestyle as an organizational goal, and improved knowledge about referral options, intervention effects, and reimbursement. Older MHPs, those who considered their lifestyle important, and those working in organizations with available interventions experienced fewer barriers.

Our findings are broadly in line with previous research in mental and general health care.^8,14,15,18^ Contrary to the barriers identified in previous studies, only few MHPs (n = 10, .7%) in this study stated that lifestyle referral was not their responsibility, a minority (n = 24, 1.9%) did not consider lifestyle important in psychiatric treatment, only two MHPs (.1%) expressed skepticism about its effectiveness. None of the MHPs were concerned about adverse effects. Regarding facilitators, only one MHP (.1%) reported that less administrative work would enable referral. These differences might be attributed to the increased attention to lifestyle psychiatry in recent years in The Netherlands.

Interestingly, we found that only a few MHPs perceived their lifestyle habits as a barrier for taking a lifestyle history and referral (3.1%, n = 47 | 1.7%, n = 21), while previous research in this study population showed that MHPs who consider their lifestyle less important and who are physically inactive take fewer lifestyle histories and refer less.^9^ MHPs might be unaware of how their lifestyle influencing their clinical lifestyle practices, and MHPs that are actively working on their lifestyle might be more inclined to promote this to patients due to their personal interest.

## Implications for Clinical Practice

The identified barriers and facilitators provide opportunities for solutions to improve MHPs’ clinical lifestyle practices and improve the implementation of lifestyle interventions for better mental and physical health outcomes in patients. In the short term, organizations can provide a better overview of available lifestyle interventions and their reimbursement, as well as improve collaboration with network partners to enhance the accessibility of these interventions. Staff should also receive education on the effects of lifestyle interventions and how to effectively discuss lifestyle changes and motivate patients.

Moreover, organizations should prioritize lifestyle by integrating it into clinical routines and treatment protocols, which can be achieved by providing a structured method for taking a lifestyle history and lifestyle referral.

Additionally, organizations should increase the availability of interventions. While smaller organizations may face challenges in this regard, they could collaborate with nearby gyms, physiotherapists, and dietitians, providing them with training on working with individuals with mental illness. In the long term, mental health organizations would benefit from enhanced collaboration across disciplines, integrating diverse team members such as sports coaches, physiotherapists, and dieticians.

Next to these organizational solutions, there are also societal challenges that need to be addressed. Lifestyle should be given a more central role within health care and society to foster environments that promote healthy living. Governmental action is needed to provide insurers with financial incentives for disease prevention. This includes better reimbursement and increased availability of lifestyle interventions that are fitted to the needs of people with mental illness.

### Strengths and Weaknesses

The strengths of this study are the large sample size and the diversity of participants, which make the results generalizable to a range of clinical settings in mental health care. Weaknesses are that there might have been selection bias, since MHPs who are interested in lifestyle might be more willing to fill out the survey. To mitigate this bias, we explicitly stating in the survey invitation that all MHPs were invited to participate, regardless of their views on in mental treatment. Additionally, we distributed the questionnaire to all mental health organizations in The Netherlands.

## Conclusions

MHPs believe that lifestyle should be part of every psychiatric treatment, yet the majority experience barriers for taking a lifestyle history and referring patients to lifestyle interventions. Older MHPs, those who consider lifestyle important, and those working in organizations with available lifestyle interventions experience fewer barriers. Organizations should prioritize lifestyle psychiatry by educating their staff, integrating it into clinical routines, and increasing the availability of interventions.

## Supplemental Material

Supplemental Material - Barriers and Facilitators of Taking a Lifestyle History and Referral to Lifestyle Interventions in Mental Health Supplemental Material for Barriers and Facilitators of Taking a Lifestyle History and Referral to Lifestyle Interventions in Mental Health by L.E.M. Koomen, J. Deenik, and W. Cahn in American Journal of Lifestyle Medicine.

Supplemental Material - Barriers and Facilitators of Taking a Lifestyle History and Referral to Lifestyle Interventions in Mental Health Supplemental Material for Barriers and Facilitators of Taking a Lifestyle History and Referral to Lifestyle Interventions in Mental Health by L.E.M. Koomen, J. Deenik, and W. Cahn in American Journal of Lifestyle Medicine.
