# Supplementary material for: Barriers and Facilitators of Taking a Lifestyle History and Referral to Lifestyle Interventions in Mental Health
Source: Am J Lifestyle Med. 2024 Jun 13:15598276241261670. Online ahead of print. doi: 10.1177/15598276241261670 (PMC11562272; doi:10.1177/15598276241261670)
Supplement: Supplemental Material - Barriers and Facilitators of Taking a Lifestyle History and Referral to Lifestyle Interventions in Mental Health [file sj-pdf-1-ajl-10.1177_15598276241261670.pdf]

Letter of certification

Amsterdam, 20th of May 2024

To whom it may concern,

The manuscript entitled 'Barriers and facilitators of taking a lifestyle history and referral to lifestyle interventions in mental health' was edited by Taalcentrum VU.

Kind regards,

Voske op het Veld

Project coordinator translations

TAALCENTRUM - VU
Laat woorden werken.

Vertalingen | Trainingen | Tekstredactie

Postadres	Bezoekadres
De Boelelaan 1105	De Boelelaan 1077
1081 HV Amsterdam	1081 HV Amsterdam
