# Supplementary material for: Barriers and Facilitators of Taking a Lifestyle History and Referral to Lifestyle Interventions in Mental Health
Source: Am J Lifestyle Med. 2024 Jun 13:15598276241261670. Online ahead of print. doi: 10.1177/15598276241261670 (PMC11562272; doi:10.1177/15598276241261670)
Supplement: Supplemental Material - Barriers and Facilitators of Taking a Lifestyle History and Referral to Lifestyle Interventions in Mental Health [file sj-pdf-2-ajl-10.1177_15598276241261670.pdf]

Appendix

Supplementary material

Survey

1. What is your age?
2. What is your gender?
   A. Male. B. Female. C. Other
3. What is your profession?

   A. General practice-based nurse specialist. B. Specialized nurse. C. Psychologist. D. Healthcare psychologist. E. Clinical psychologist. F. Psychiatry resident. G. Psychiatrist. H. Other → If other, what is your profession? (open question)

4. Where do you mainly work?
   A. Organization for psychiatric care. B. Hospital. C. Academic hospital. D. Own practice. E. General practitioners practice. F. Other → If other, where do you work? (open question)
5. What is your main population?
   A. Children/youth. B. Adults. C. Elderly. D. Not specifically one population
6. Are lifestyle interventions available within your work setting?
   A. If yes: what kind of lifestyle interventions?
7. What is your length?
8. What is your weight?
9. Do you smoke?
   A. Yes. B. No. C. I have smoked in the past
10. Do you drink alcohol?
    A. Yes. B. No
11. Do you use drugs?
    A. Yes
       - If yes: How often do you use drugs?
         1. Daily. 2. Weekly. 3. Monthly. 4. Several time a year. 5. Yearly. 6. Less than yearly

    B. No
12. How important do you find your own lifestyle on a scale of 0-10?
13. How many days per week on average do you do physical activity of medium or high intensity (with increased heart rate/respiration, e.g. brisk walking, cycling, team sports)?
14. What kind of physical activities do you do?
    A. Walking. B. Bicycling. C. Running. D. Cycling. E. Gym. F. Team sports. G. Tennis. H. Golf. I. Boxing. J. Swimming. K. Crossfit. L. Other → If other, what? (open question)
15. How many minutes per week do you spend on these activities? (open question)
16. How many days per week on average do you do muscle and bone strengthening exercises? (0-7)
17. Do you have a balanced eating pattern?
    A. Yes. B. No
18. How satisfied are you with your sleep on a scale of 0-10 (0 = very unsatisfied, 10 = very satisfied)?
19. How much stress do you experience at work on a scale of 0-10 (0 = no stress, 10 = a lot of stress)?
20. How much stress do you experience at home on a scale of 0-10 (0 = no stress, 10 = a lot of stress)?
21. How important do you find lifestyle in psychiatric treatment?
    A. Scale 0-10 (0 = not important at all, 10 = very important)
22. Statement: lifestyle should be part of any psychiatric treatment
    A. Totally disagree. B. Disagree. C. Neutral. D. Agree. E. Totally agree
23. What could help you to discuss lifestyle with a patient?

A. More time
B. More knowledge on how to discuss the topic lifestyle
C. More knowledge on the effect of lifestyle on mental health
D. More knowledge on referral possibilities
E. A tool (e.g. questionnaire, app)
F. Embedding of lifestyle in daily working routine
G. Support of colleagues
H. It would help if the patient would ask for
I. It would help if it is an organization's goal
J. I do not think it is necessary to discuss it more often

24. Are there other things you could help you to discuss lifestyle with a patient?
    A. Yes → If yes: What? (open question)
    B. No
25. What hinders you from discussing lifestyle with a patient?
    A. Lack of time
    B. Lack of knowledge on lifestyle
    C. Lack of knowledge on discussing the topic lifestyle
    D. Lack of knowledge on the effect of lifestyle on mental health
    E. Lack of knowledge on referral possibilities
    F. Patients do not find it important
    G. My own lifestyle habits
    H. My organization does not find it important
    I. It is too chaotic in my organization
    J. Lack of support of colleagues
    K. I do not find lifestyle important in psychiatric treatment
    L. I do not experience any barriers
26. Are there other things that hinder you to discuss lifestyle with a patient?
    A. Yes → If yes: What? (open question)
    B. No
27. What could help you to refer patients to lifestyle interventions?
    A. More time
    B. More knowledge on the effect of lifestyle on mental health
    C. More knowledge on referral possibilities
    D. More knowledge on reimbursement of lifestyle interventions
    E. More referral possibilities within my organization
    F. More referral possibilities within the region
    G. Experience with positive effects in patients
    H. It would help if lifestyle interventions are standard in the treatment within my organization
    I. I do not find it important to refer to lifestyle interventions
28. Are there other things you could help you to refer patients to lifestyle interventions?
    A. Yes → If yes: What? (open question)
    B. No
29. What hinders you from referring patients to lifestyle interventions?
    A. Lack of time
    B. Lack of knowledge on the effect of lifestyle on mental health
    C. Lack of knowledge on referral possibilities
    D. Lack of knowledge on reimbursement of lifestyle interventions
    E. Lack of referral possibilities within my organization
    F. Lack of referral possibilities within the region
    G. Lifestyle is not seen as part of psychiatric treatment in my organization
    H. My own lifestyle habits
    I. It is too chaotic in my organization
    J. I do not find lifestyle important in psychiatric treatment

K. I do not experience any barriers

30. Are there other things that hinder you to hinder you to refer patients to lifestyle interventions?
    A. Yes → If yes: What? (open question)
    B. No

Variable	Mean or number (SD or %)
Number of participants	1524.0
Age	42.2 (11.3)
Female gender	1198.0 (78.6)
Profession	
Other	89.0 (5.8)
Psychologist	168.0 (11.0)
Resident in psychiatry	156.0 (10.2)
GP-based nurse specialist	191.0 (12.5)
Referring MHP with nursing background	225.0 14.8)
Referring MHP with scientific background	329.0 (21.6)
Clinical psychologist	81.0 (5.3)
Psychiatrist	285.0 (18.7)
Work setting	
Organization offering specialized mental healthcare	1111.0 (72.9)
Hospital	47.0 (3.1)
Academic hospital	95.0 (6.2)
Own practice	41.0 (2.7)
General practitioner's practice	183.0 (12.0)
Other	46.0 (3.0)
Main population, n (%)	
Children/youth	285.0 (18.7)
Adults	1004.0 (65.9)
Elderly	81.0 (5.3)
Not specifically one population	153.0 (10.0)
Lifestyle interventions available within work setting	965.0 (63.3)
BMI, mean (SD)	23.9 (3.6)
BMI >24.9	464.0 (30.4)
Current smoker	80.0 (5.2)
Smoked in past	201.0 (13.2)
Drinks alcohol	1109.0 (72.8)
Uses drugs	104.0 (6.8)
Adheres to Dutch physical activity norm	702.0 (46.1)

Table S2. Demographics and information on lifestyle habits of the mental health professionals. BMI=body mass index, GP=general practitioner, MHP=mental health professional, N=number, SD=standard deviation.

Barriers taking a lifestyle history	Frequency (n=207) (%)	Facilitators taking a lifestyle history	Frequency (n=252) (%)
Overshadowing of patient's mental problems	52 (25.1)	More lifestyle interventions at organization	45 (17.9)
I do not think about it to discuss it/lack of awareness	26 (12.6)	Structured method of taking a lifestyle history. Diagnostics, treatment and incorporation in staff meetings	45 (17.9)
Lack of lifestyle interventions at organization	19 (9.2)	More priority and support on lifestyle by colleagues, supervisors and organization	22 (8.7)
Not incorporated in daily practice, protocols	14 (6.8)	Education on lifestyle	19 (7.5)
Patients do not ask for it	14 (6.8)	Better patient education	18 (7.1)
Lifestyle interventions are too patronizing/I am afraid to be to patronizing	12 (5.8)	More accessible interventions in region with good interdisciplinary collaboration and overview of these interventions	17 (6.8)
Organization does not prioritize lifestyle	10 (4.8)	Better collaboration with GP, and other network partners as municipality, and insurers	16 (6.4)
Patients are not motivated	9 (4.4)	Better facilities in organization	11 (4.4)
Treatments are too symptom orientated, and not holistic	9 (4.4)	Better incorporation of eHealth on lifestyle	11 (4.4)
Lack of time	8 (3.9)	Societal priority on lifestyle	8 (3.2)
Lack of knowledge about how to discuss lifestyle, and the effect of lifestyle interventions	7 (3.4)	Reimbursement for lifestyle interventions in psychiatry	8 (3.2)
It takes a lot of time, and results of lifestyle changes are limited	7 (3.4)	More time	7 (2.8)
The medical doctor/psychiatrist should give lifestyle advice	6 (2.9)	More knowledge on interventions in region	7 (2.8)
Daily practice is too hectic	6 (2.9)	Improve own lifestyle. and set a good example for patients	6 (2.4)
Other physicians/colleagues do not prioritize lifestyle	4 (1.9)	A dedicated tool	4 (1.6)
Unhealthy living environment of patients	3 (1.5)	Better guidelines	4 (1.6)
Lack of patient education	3 (1.5)	Self-reported questionnaires about lifestyle for patients	3 (1.2)
Lack of accessible lifestyle interventions in region	3 (1.5)	Awareness from insurer	3 (1.2)
No reimbursement for lifestyle anamnesis/interventions	3 (1.5)	More scientific research on lifestyle	3 (1.2)
Patients already have to do so much	3 (1.5)	More awareness of lifestyle by patient	2 (0.8)
Lack of societal priority	3 (1.5)	More staff	2 (0.8)
I believe that patients do not want lifestyle interventions as a treatment	2 (1.0)	Support of patient's near ones	2 (0.8)
Lack of collaboration with e.g. dietician. physiotherapist	1 (0.5)	Organization's vision on smoking cessation	1 (0.4)
I found it difficult because I have to educate the children's parents	1 (0.5)	More information from GP	1 (0.4)
Lack of a tool to discuss lifestyle	1 (0.5)	Advice from GP about lifestyle	1 (0.4)
Lack of finances in organization	1 (0.5)	More priority on lifestyle instead of only focusing on mental health	1 (0.4)
I do not think I can change the lifestyle of my patient	1 (0.5)	Availability of lifestyle interventions when patients are on the waiting list	1 (0.4)
I found it difficult to discuss lifestyle with adolescents	1 (0.5)		
I think the GP should discuss it	1 (0.5)		
I work in forensics and we focus on lowering recurrence risk, and lifestyle is not a factor of recurrence risk	1 (0.5)		
I work with children below 6, and lifestyle is not suitable for them	1 (0.5)		
My weight	1 (0.5)		

I work with children and lifestyle changes are difficult for children	1 (0.5)		
Barriers referral	Frequency n=53 (%)	Facilitators referral	Frequency n=59 (%)
Lack of reimbusement for lifestyle interventions	13 (24.5)	More accessible lifestyle interventions for patient with mental illness	13 (22.0)
Lack of patient motivation	12 (22.6)	Societal priority on lifestyle	7 (11.9)
Lack of accessible lifestyle interventions for patients with mental illness	9 (17)	If the organization would prioritize it	6 (10.2)
Overshadowing of patient's mental problems	6 (11.3)	Better collaboration with GP, and other network partners as municipality, and insurers	6 (10.2)
Lack of education about motivational interviewing. knowledge on the costs and effects of lifestyle interventions	3 (5.7)	More lifestyle interventions in organization	5 (8.5)
I think the GP should do this	3 (5.7)	Education on the effect of lifestyle interventions and motivational interviewing	5 (8.5)
Referral is an administrative hassle	2 (3.8)	Overview of accessible lifestyle interventions for patients with mental illness	4 (6.8)
Lack of knowledge about accessible lifestyle interventions	2 (3.8)	More possibilities to do long-term lifestyle interventions together with patients	3 (5.1)
Patients do not understand why they are referred to lifestyle interventions	1 (1.9)	More time	2 (3.4)
Patients have side effects of psychotropic drugs that hinder participation in lifestyle interventions	1 (1.9)	If patients ask for it	2 (3.4)
Lack of collaboration with GP, and other network partners as municipality, and insurers	1 (1.9)	A dedicated lifestyle physician	2 (3.4)
I believe it is patronizing and harms the therapeutic relationship	1 (1.9)	Better embedding in daily work routine	2 (3.4)
I forget it	1 (1.9)	More focus from the GP	1 (1.7)
I do not have frequent patient contact	1 (1.9)	Patient education	1 (1.7)
Lack of societal priority	1 (1.9)	I would have to find it more interesting	1 (1.7)
It is not embedded in daily clinical care	1 (1.9)	More staff	1 (1.7)
Lack of patient education	1 (1.9)	Shorter waiting times	1 (1.7)
I am afraid that patients would think that their psychiatric complaints are their own fault if I refer them to lifestyle interventions	1 (1.9)	Less administration	1 (1.7)
Supervisor does not prioritize lifestyle	1 (1.9)	Better collaboration with patient's near ones	1 (1.7)
Waiting lists	1 (1.9)	More holistic view in treatment	1 (1.7)

Table S3. Overview of answers on barriers and facilitators of taking a lifestyle history and referral at the open-ended questions.

	Barriers for taking a lifestyle history n=1524											
	Organizational barriers			Knowledge barriers			Barrier patient disinterest			Barrier own lifestyle		
Variable	OR	CI	p	OR	CI	p	OR	CI	p	OR	CI	p
Lifestyle interventions at organization (reference = no interventions available)	0.75	0.61 - 0.93	0.007	0.65	0.53 - 0.79	<0.001	1.21	0.94 - 1.56	0.15	1.20	0.61 - 2.36	0.60
Age per 10 years	0.74	0.66 - 0.82	<0.001	0.68	0.61 - 0.76	<0.001	0.82	0.72 - 0.93	<0.001	0.59	0.40 - 0.86	0.007
Male gender	1.19	0.92 - 1.53	0.18	0.93	0.73 - 1.19	0.56	1.43	1.06 - 1.91	0.02	1.12	0.50 - 2.53	0.79
BMI per 5 units	1.01	0.87 - 1.18	0.87	1.04	0.90 - 1.20	0.60	1.23	1.04 - 1.47	0.02	2.99	2.10 - 4.27	<0.001
How important is own lifestyle	1.03	0.93 - 1.13	0.58	0.83	0.76 - 0.92	<0.001	1.00	0.89 - 1.13	0.97	0.60	0.46 - 0.80	<0.001
Smoking status (reference = smoker)												
Smoked in past	0.78	0.47 - 1.28	0.33	0.90	0.56 - 1.47	0.69	0.62	0.35 - 1.08	0.09	0.36	0.10 - 1.27	0.11
Never smoked	0.72	0.47 - 1.12	0.15	0.85	0.56 - 1.30	0.45	0.52	0.32 - 0.85	0.01	0.37	0.13 - 1.04	0.06
No alcohol use (reference = yes)	1.00	0.80 - 1.25	0.97	1.03	0.83 - 1.28	0.79	0.97	0.74 - 1.27	0.81	1.07	0.52 - 2.17	0.86
Physical activity (reference = meets no criteria)												
Meets 2 criteria	1.48	1.13 - 1.94	0.005	0.75	0.58 - 0.98	0.03	1.07	0.77 - 1.47	0.70	0.33	0.14 - 0.79	0.01
Meets 1 criterium	1.39	1.05 - 1.84	0.02	0.76	0.58 - 0.99	0.04	0.83	0.59 - 1.17	0.28	0.61	0.29 - 1.29	0.20
Dietary habits (reference = unbalanced eat pattern)	0.88	0.64 - 1.21	0.43	0.94	0.69 - 1.27	0.68	0.92	0.64 - 1.33	0.66	0.47	0.23 - 0.94	0.03
Sleep satisfaction	0.92	0.86 - 0.97	<0.001	0.98	0.93 - 1.04	0.57	0.99	0.92 - 1.07	0.86	0.91	0.77 - 1.08	0.27
Profession (reference = psychiatrist)												
Other	0.67	0.42 - 1.08	0.20	1.09	0.69 - 1.73	0.72	1.57	0.92 - 2.66	0.10	0.56	0.11 - 2.90	0.49
Psychologist	0.50	0.33 - 0.76	<0.001	1.61	1.08 - 2.39	0.02	0.81	0.49 - 1.33	0.40	0.20	0.02 - 1.89	0.16
Resident in psychiatry	1.29	0.85 - 1.95	0.23	1.57	1.06 - 2.33	0.03	1.01	0.62 - 1.63	0.98	0.98	0.26 - 3.68	0.97
GP-based nurse specialist	0.36	0.25 - 0.53	<0.001	0.85	0.60 - 1.22	0.38	1.14	0.73 - 1.76	0.57	1.90	0.59 - 6.10	0.28
Referring MHP with nursing background	0.70	0.49 - 0.99	0.04	0.58	0.41 - 0.82	<0.001	1.12	0.75 - 1.68	0.59	1.03	0.33 - 3.23	0.96
Referring MHP with scientific background	0.54	0.39 - 0.75	<0.001	1.26	0.92 - 1.72	0.15	0.68	0.45 - 1.02	0.06	1.35	0.45 - 4.05	0.59
Clinical psychologist	0.67	0.42 - 1.08	0.10	1.42	0.90 - 2.22	0.13	0.59	0.30 - 1.13	0.11	1.13	0.20 - 6.23	0.89

Table S4. Results of the linear regression analyses on barrier categories for taking a lifestyle history. Organizational barriers= lack of time, 'it is too chaotic in my organization', 'my organization does not find it important', lack of support of colleagues. Knowledge barriers= lack of knowledge about referral possibilities, lack of knowledge about the effect of lifestyle on mental health, lack of knowledge about lifestyle, lack of knowledge about discussing the topic lifestyle, 'I feel uncomfortable to discuss the lifestyle of my patients'. BMI=body mass index, CI=confidence interval, GP=general practitioner, MHP=mental health professional, n=number, OR=odds ratio, p=p-value.

	Barriers for referral to lifestyle interventions N=1263								
	Organizational barriers			Knowledge barriers			Own lifestyle		
Variable	OR	CI	p	OR	CI	p	OR	CI	p
Lifestyle interventions at organization (reference = no interventions available)	0.85	0.68 - 1.06	0.15	0.68	0.55 - 0.85	<0.001	0.74	0.28 - 1.95	0.54
Age per 10 years	0.77	0.69 - 0.87	<0.001	0.73	0.65 - 0.81	<0.001	0.49	0.28 - 0.88	0.02
Male gender	1.08	0.84 - 1.38	0.56	0.88	0.69 - 1.14	0.33	1.45	0.49 - 4.32	0.50
BMI per 5 units	0.91	0.78 - 1.07	0.26	0.95	0.82 - 1.11	0.54	2.49	1.46 - 4.25	<0.001
How important is own lifestyle	1.00	0.90 - 1.10	0.95	0.95	0.86 - 1.05	0.30	0.48	0.31 - 0.72	<0.001
Smoking status (reference = smoker)									
Smoked in past	0.68	0.40 - 1.17	0.16	1.10	0.64 - 1.90	0.72	0.26	0.06 - 1.16	0.08
Never smoked	0.66	0.41 - 1.07	0.09	0.96	0.59 - 1.56	0.86	0.11	0.03 - 0.43	<0.001
Alcohol use (reference = yes)	0.94	0.74 - 1.19	0.60	0.84	0.67 - 1.06	0.15	0.75	0.23 - 2.44	0.63
Physical activity (reference = meets no criteria)									
Meets 2 criteria	1.02	0.77 - 1.36	0.89	0.79	0.60 - 1.04	0.09	0.18	0.04 - 0.84	0.03
Meets 1 criterium	1.21	0.91 - 1.61	0.20	0.79	0.59 - 1.04	0.09	0.35	0.11 - 1.12	0.08
Dietary habits (reference = unbalanced eat pattern)	0.93	0.67 - 1.30	0.68	0.85	0.61 - 1.17	0.32	1.66	0.46 - 5.95	0.44
Sleep satisfaction	0.98	0.92 - 1.05	0.62	0.95	0.89 - 1.01	0.12	0.99	0.75 - 1.30	0.94
Profession (reference = psychiatrist)									
Resident in psychiatry	0.91	0.61 - 1.35	0.64	1.24	0.83 - 1.85	0.29	0.29	0.06 - 1.50	0.14
GP-based nurse specialist	0.32	0.22 - 0.45	<0.001	1.01	0.72 - 1.44	0.94	0.78	0.20 - 3.09	0.73
Referring MHP with nursing background	0.71	0.51 - 0.99	0.04	0.96	0.69 - 1.34	0.81	0.15	0.03 - 0.83	0.03

Referring MHP with scientific background	0.51	0.37 - 0.70	<0.001	1.07	0.79 - 1.46	0.67	0.15	0.03 - 0.85	0.03
Clinical psychologist	0.81	0.52 - 1.26	0.35	1.27	0.81 - 2.00	0.30	0.64	0.10 - 4.04	0.64

Table S5. Results of the linear regression analyses on barrier categories of referral to lifestyle interventions. Organizational barriers= lack of referral possibilities within my organization, lack of time, lack of referral possibilities within the region, lifestyle is not seen as part of psychiatric treatment in my organization, 'it is too chaotic in my organization'. Knowledge barriers= lack of knowledge about referral possibilities, lack of knowledge about reimbursement of lifestyle interventions, lack of knowledge about the effect of lifestyle on mental health. B=coefficient, BMI=body mass index, CI=confidence interval, GP=general practitioner, MHP=mental health professional, n=number, OR=odds ratio, p=p-value.
